# Individual differences in classification images of Mooney faces

**DOI:** 10.1167/jov.22.13.3

**Published:** 2022-12-02

**Authors:** Teresa Canas-Bajo, David Whitney

**Affiliations:** 1 Vision Science Graduate Group, University of California, Berkeley, Berkeley, CA, USA; 2 Department of Psychology, University of California, Berkeley, Berkeley, CA, USA

**Keywords:** individual differences, holistic perception, face recognition, classification images

## Abstract

Human face recognition is robust even under conditions of extreme lighting and in situations where there is high noise and uncertainty. Mooney faces are a canonical example of this: Mooney faces are two-tone shadow-defined images that are readily and holistically recognized despite lacking easily segmented face features. Face perception in such impoverished situations—and Mooney face perception in particular—is often thought to be supported by comparing encountered faces to stored templates. Here, we used a classification image approach to measure the templates that observers use to recognize Mooney faces. Visualizing these templates reveals the regions and structures of the image that best predict individual observer recognition, and they reflect the underlying internal representation of faces. Using this approach, we tested whether there are classification images that are consistent from session to session, whether the classification images are observer-specific, and whether they allow for pattern completion of holistic representations even in the absence of an underlying signal. We found that classification images of Mooney faces were indeed non-random (i.e., consistent session from session) within each observer, but they were different between observers. This result is in line with previously proposed existence of face templates that support face recognition, and further suggests that these templates may be unique to each observer and could drive idiosyncratic individual differences in holistic face recognition. Moreover, we found classification images that reflected information within the blank regions of the original Mooney faces, suggesting that observers may fill in missing information using idiosyncratic internal information about faces.

## Introduction

Faces play a central role in our everyday life, and our visual system is in turn extremely sensitive to them. Faces convey critical social and emotional information; they guide interactions and our everyday behavior (Costela & Woods, 2019; Jack & Schyns, 2015; Coutrot & Guyader, 2014). Reinforced by a lifelong exposure to faces, humans have developed a remarkable preference for faces over other objects from infancy (Umiltà, Simion & Valenza, 1996; Farzin, Rivera & Whitney, 2009; Frank, Vul & Johnson, 2009; Pascalis & Kelly, 2009). We quickly find faces in a scene and direct our attention and gaze toward them (Bindemann, Burton, Hooge, Jenkins & de Haan, 2005; Boucart, Lenoble, Quettelart, Szaffarczyk, Despretz, & Thorpe, 2016; Cerf, Frady & Koch, 2009; Cerf, Harel, Huth, Einhauser & Koch, 2008; Costela & Woods, 2019; Coutrot & Guyader, 2014; Crouzet, Kirchner & Thorpe, 2010; Foulsham, Cheng, Tracy, Henrich & Kingstone, 2010; Jack & Schyns, 2015; Marat, Rahman, Pellerin, Guyader & Houzet. 2013; Martin, Davis, Riesenhuber., & Thorpe, 2018; Ro, Russell & Lavie, 2001; Theeuwes & Van der Stigchel, 2006; Wolfe & Whitney, 2015; Xia, Manassi, Nakayama, Zipser & Whitney, 2020). In fact, humans can recognize faces under a wide range of conditions, including under challenging environments (Burton, Bruce & Hancock, 1999; Hole, George, Eaves & Rasek, 2002). Humans have also developed expert processing mechanisms specific to faces such as holistic processing, the perception of faces as a whole (Farah, Wilson, Drain & Tanaka, 1998; Maurer, Le Grand & Mondloch, 2002; Sergent, 1984). Faces therefore enjoy a unique and privileged position in human visual processing.

**Figure 1.**
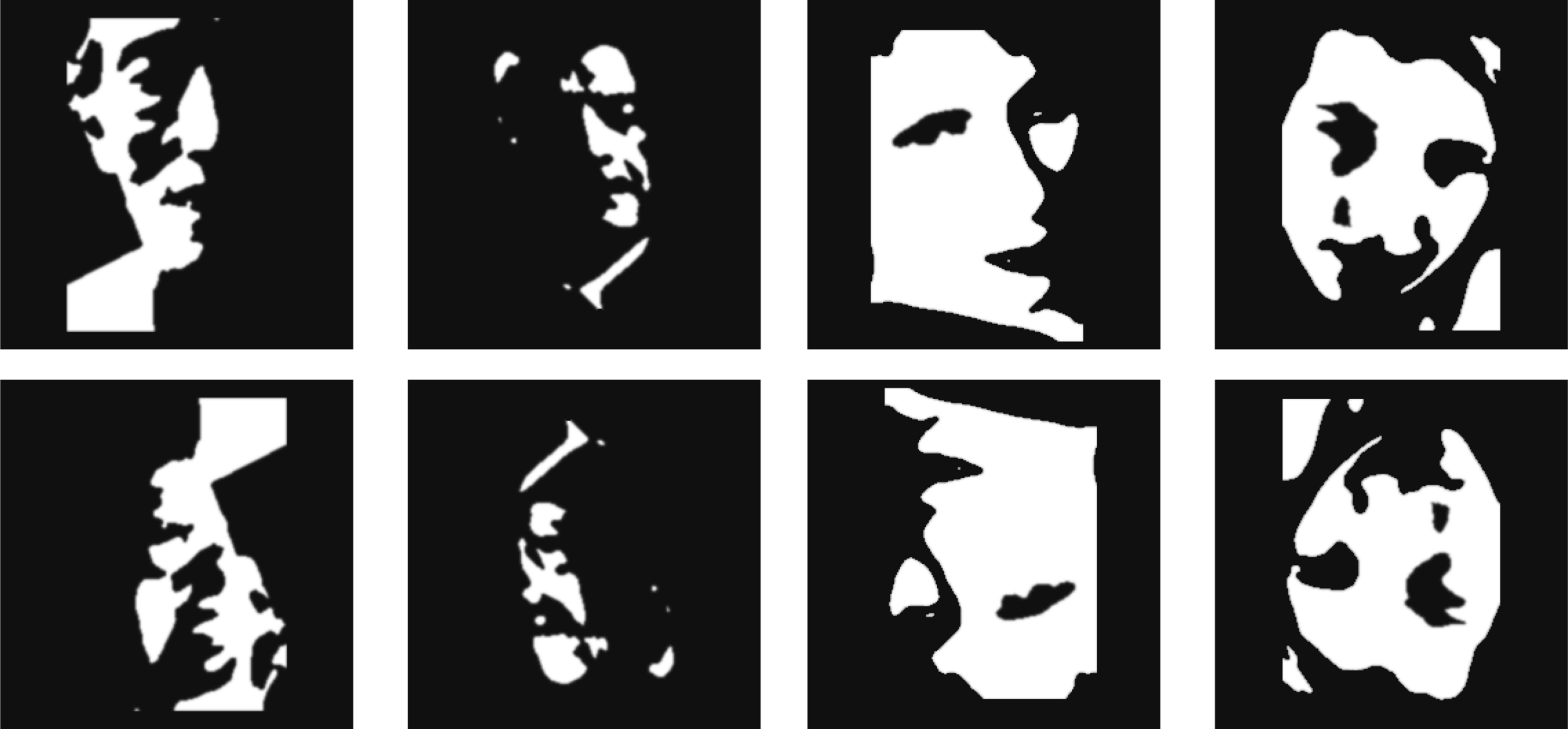
Mooney faces used in this study in upright (top) and inverted (bottom) orientation. Mooney faces are two-tone images that can be quickly recognized as faces despite lacking low-level face features. Mooney faces are processed as a whole; note that although upright Mooney faces are easily perceived as faces, inverted Mooney faces are difficult or even impossible to recognize as such. For this reason, Mooney faces are ideal stimuli to test holistic processing.

Previous work suggests that face recognition may involve comparing encountered faces to internal face representations – templates (Cavanagh, 1991; Damasio, Damasio & Van Hoesen, 1982; Sekuler & Abrams, 1968; Sergent, 1984; Simpson & Crandall, 1972; Smith & Nielsen, 1970; Valentine, 1991). Faces that best match those templates are processed more efficiently than those that do not (Rossion, 2008; Rossion & Boremanse, 2008). This matching process allows the recovery of two- and three-dimensional information from the observed faces, even in impoverished or extreme lighting conditions (Cavanagh, 1991). Valentine (1991) defined the norm-based coding model, which proposes that faces are encoded as distance vectors to a prototype shaped by individual prior experience. Others have aimed to define the structures of these templates and proposed similar models of face recognition that occur by matching observed faces to template-like structures (e.g., Bar codes: Dakin & Watt, 2009; Integral Images: Viola & Jones, 2004; Face Recognition Units: Bruce & Young, 1986; Young & Bruce, 2011). In these models, faces can be encoded in terms of low-level features that can be parsed or easily segmented. Although these models can accommodate grayscale faces well, they may not be as effective for faces that require holistic processing. For example, they would not be as successful for two-tone shadow defined faces (Mooney faces; Figure 1) because the local features, edges, contours, and spatial frequency content of Mooney faces are not discriminative of the image as a face (Andrews & Schluppeck, 2004; Bona, Cattaneo & Silvanto, 2016; Cavanagh, 1991; Farzin et al., 2009; Latinus & Taylor, 2005; McKone, 2004; Moore & Cavanagh, 1998). In principle, template-based models are an appropriate way to analyze Mooney faces (Cavanagh, 1991), but the templates need to carry information about the holistic aspects of the face.

Previous work has shown that there are individual differences in holistic processing of both grayscale and two-tone Mooney faces: some individuals process faces more holistically than others (DeGutis, Mercado, Wilmer, & Rosenblatt, 2013; DeGutis, Wilmer, Mercado, Cohan, 2013; Gauthier, 2020; Russell, Duchaine & Nakayama, 2009; Wang, Li, Fang, Tian & Liu, 2012). Interestingly, there are also stimulus-specific individual differences in holistic processing (Canas-Bajo & Whitney, 2020): specific faces that are processed holistically by one observer are not necessarily processed holistically by other observers. The origin of these differences remains unclear. Here, our goal was to test whether such idiosyncrasy in holistic processing is caused by underlying individual differences in observer-specific face templates.

Capturing or visualizing templates can be challenging because they are implicit representations. One of the most widely used and established techniques for measuring templates is the reverse-correlation image classification method (see Murray, 2011 for a review). This technique is a data-driven method that has been widely used in the literature to capture internal representations—the image regions and structures that are most important for detection, discrimination, or classification (Ahumada, 1996, 2002; Ahumada & Lovell, 1971; Beard & Ahumada, 1998; Mareschal, Dakin & Bex, 2006; Neri & Heeger ,2002; Ringach & Shapley, 2004; Solomon, 2002; Victor, 2005; Watson, 1998). More recently, the method has also been used to visualize face templates (Brinkman, Todorov & Dostch, 2017; Dostch & Todorov, 2012; Dotsch, Wigboldus, Langner & van Knippenberg, 2008; Éthier-Majcher, Joubert & Gosselin, 2013; Gosselin and Schyns, 2003; Karremans, Dotsch, & Corneille, 2011). In a standard reverse-correlation experiment, the stimuli consist of random noise overlaid over the same base face. The observers’ task is to classify the stimuli based on some construct (e.g., trustworthiness: Éthier-Majcher, Joubert & Gosselin, 2013). Classification images are computed by averaging the noise patterns of the stimuli that participants classified as being representative of the construct of interest. Classification images can therefore serve as a visualization of internal representations of faces (Brinkman, Todorov & Dostch, 2017; Dostch & Todorov, 2012; Gosselin & Schyns, 2003; Sekuler, Gaspar, Gold & Bennett, 2004). The reverse-correlation technique is ideal to tap into implicit representations of faces because it is a purely data-driven method: noise is generated randomly in each trial for each participant, and participants use their own internal representation of the construct without external biases (Mangini & Biederman, 2004; Todorov, Dotsch, Wigboldus, & Said, 2011). In the field of face recognition, this method has been used to understand the basis of face recognition at the group level (Mangini & Biederman, 2004; Sekuler et al., 2004) and how it is influenced by factors like emotion (Brooks & Freeman, 2018; Karremans, Dostch & Corneille, 2011; Jack, Caldara & Schyns, 2012), perceptual disorders (Brinkman, Dotsch, Zondergeld, Koevoets, Aarts & van Haren, 2019), prejudice (Dotsch, Wigboldus, & Van Knippenberg, 2013), social cognition (Brooks, Stolier & Freeman, 2018), and culture (Dotsch et al., 2008; Jack et al., 2012; Ratner, Dotsch, Wigboldus, van Knippenberg, & Amodio, 2014). For instance, Dotsch and Todorov (2012) used classification images to identify racial biases in the perception of trustworthiness in faces.

Here, we use the reverse correlation technique to investigate whether there are individual differences in classification images of Mooney faces. Mooney faces are highly impoverished two-tone black and white blobs that are readily perceived as faces despite lacking low-level face features that can be parsed in a bottom-up fashion (Figure 1; Brodski, Paasch, Helbling & Wibral, 2015; Goold & Meng, 2016; Ke, Stella, & Whitney, 2017; Mooney, 1957; Schwiedrzik, Melloni & Schurger, 2018). That is, the image must be recognized as a face before any feature (e.g., an eye, a mouth, etc) can be identified or localized (Cavanagh, 1991; Farzin et al., 2009). Consequently, holistic processing plays a critical role in the recognition of Mooney faces. Holistic processing breaks down with inverted faces, which makes inverted Mooney faces very difficult to recognize (Andrews & Schluppeck, 2004; Bona, Cattaneo & Silvanto, 2016; Cavanagh, 1991; Farzin et al., 2009; Latinus & Taylor, 2005; McKone, 2004; Moore & Cavanagh, 1998; Sergent, 1984). For these reasons, Mooney faces are ideal stimuli to isolate holistic processing (Figure 1).

The following experiments tested several hypotheses. First, because our goal was to measure the templates that support holistic perception of Mooney faces, we expected that classification images would be consistent from day to day. Second, if previously found individual differences in holistic processing reflect underlying observer-specific templates (Canas-Bajo & Whitney, 2020), we should find idiosyncratic classification images only for upright but not inverted Mooney faces. Last, if Mooney faces are recognized by matching the stimuli to stored templates, classification images of Mooney faces should reflect filled-in information that is missing in the original, impoverished stimuli. Altogether, our study aimed to investigate whether there are observer-specific face templates (Experiment 1) and whether they allow for pattern completion of the missing information in the original Mooney faces (Experiment 2).

## Experiment 1

### Methods

#### Participants

Seven participants (three male, four female) took part in this experiment. One participant was excluded because they did not complete all trials of the experiment. All subjects were recruited through Mechanical Turk and provided written consent forms before participation. All experimental procedures were approved by the UC Berkeley Institutional Review Board.

#### Material and design

The base faces in the reverse correlation task consisted of four unfamiliar Mooney faces created by Schwiedrzik, Melloni & Schurger (2018; face labels in the original dataset: U0034, U0129, U0159, U0393). These Mooney faces were presented both in upright and inverted orientations, generating a total of eight base faces for the reverse correlation experiment (Figure 1). In order to assess the consistency of the subjects’ classification images, participants did the experiment twice in two separate days. The time interval between the two sessions was on average 3.4 days (std: 1.5 days). In each experimental session, participants completed 8 blocks of 250 trials for each base image. Therefore each participant completed 4000 trials in total: base images × 250 trials per base image × 2 sessions (2000 trials per session).

The presentation of the stimuli and the data collection was controlled using Qualtrics (https://www.qualtrics.com). Note that participants used different monitors, so distance to monitor could not be controlled.

#### Procedure

**Figure 2.**
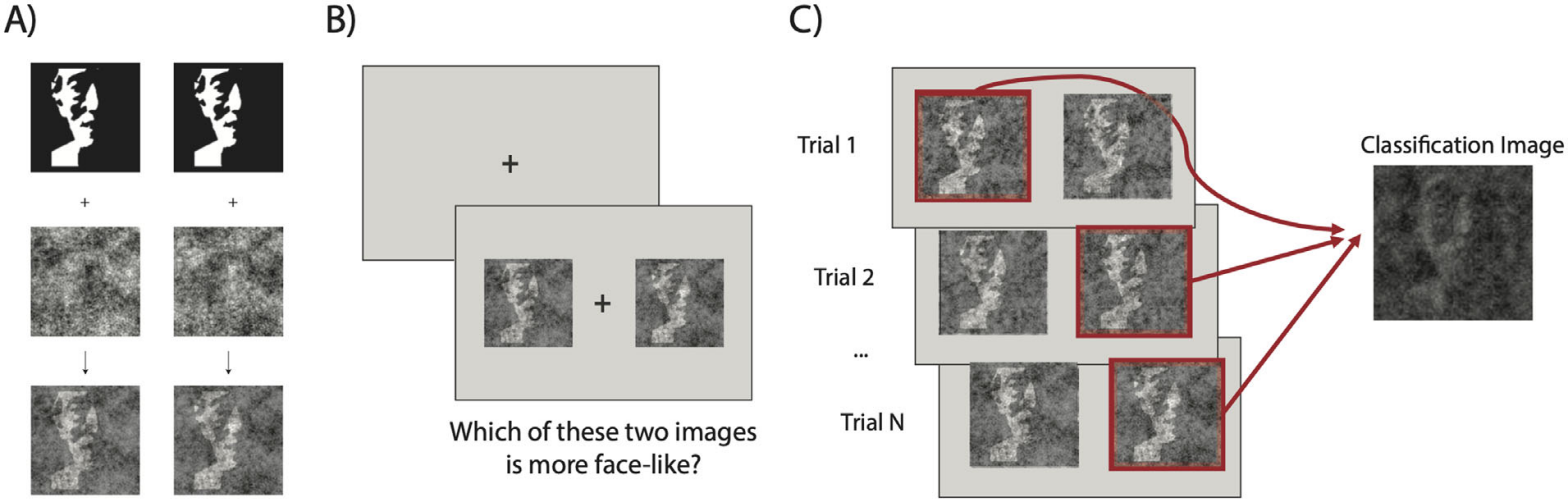
(A) To generate the stimuli for each trial, the same base image was overlaid with two identical but polarity-reversed random patterns of noise. (B) Example trial. After a blank screen, two stimuli were presented, and participants were asked to select which of two images was more face-like. (C) Classification images were generated by averaging the selected noise patterns across trials.

Stimuli were generated uniquely for each participant. In each trial, we generated a random pattern of sinusoidal noise and its corresponding negative version (Figure 2A). The random sinusoidal noise is defined by 4092 parameters, each defining the amplitude of one truncated sinusoid ranging over two cycles (for more details on how the random noise was generated, see Dostch & Todorov, 2012).

Each trial consisted of two side-by-side stimuli: the base face with the original noise superimposed and the same base face with the polarity reversed noise superimposed (Figure 2C). Participants’ task was to select which of the two images presented was more face-like by pressing the left or right button. The stimuli were presented until observers gave a response. Participants could move their eyes freely during the trial and could take a break every 250 trials. The patterns of noise were randomized for each trial separately for each observer. That is, no two subjects saw the same stimuli, and the same observer did not see the same stimuli more than once. All participants completed a demographics questionnaire followed by a question regarding their previous experience with Mooney faces.

#### Data analysis

Our goal here was to investigate whether face templates support recognition of Mooney faces, and whether the templates, if present, are observer-specific. We used classification images to visualize observers’ face templates. To generate the classification images, we averaged all of the observer's selected noise patterns across trials, and we superimposed the resulting noise on the base face (Figure 2C). The classification image reveals the regions or structures that observers rely on to classify an image as face-like. We generated one classification image per base face, per subject. The outcome was eight unique classification images for each observer.

To evaluate whether observers’ classification images were nonrandom, we tested the day-to-day consistency of each observers’ classification images for each of the eight base Mooney faces. The within-subject consistency was defined as the test-retest pixel-wise correlation between an observer's classification images in the first and second session. We then averaged all within-observer Fisher z transformed correlations for upright and inverted base faces, separately. Second, we quantified the between-observer agreement in classification images of Mooney faces. To this end, we calculated the between-observer correlations across different observers’ classification images for each base face. Then we averaged all between-observer Fisher *z* transformed correlations for upright and inverted base faces, separately.

To test the significance of the within- and between-subject correlations, we calculated a null distribution of permuted correlations. In each iteration of the permutation, for each base face, we shuffled the responses that were given by each participant. We then calculated a classification image from these shuffled responses. This was effectively the classification images an observer would have if the participant responded randomly. Using this data, we generated a set of 1000 permuted classification images, per base face, per participant. Note that our classification images have signal included, which could artificially inflate the correlations in both within- and between-subject analyses. To control for this, we calculated null distributions of permuted correlations, which maintain all of the same signal information, but represent shuffled responses, so any inflation of the correlations will occur in the null distribution as well. In other words, we never compare correlations to zero, always to permuted shuffled null distributions. To generate the distribution of permuted null within-subject correlations, for each observer and each base face, we correlated the empirical classification image of the first session with each of the permuted null classification images of the second session. In the same fashion, to generate the distribution of permuted null between-subject correlations, for each pair observers and each base face, we correlated an observer's one-session empirical classification image with each of the permuted null classification images of another observer's session.

Last, we quantified the individual differences of classification images of Mooney faces by comparing within- and between-observer agreement. The significance of this comparison was tested using a nonparametric permutation method: we computed the empirical difference for within- and between-correlations and compared it to the null difference for the between and within correlations for randomized responses. Across all analyses, to calculate the average within- and between-subject correlations across base faces or across observers, correlations were first transformed from Pearson *R* correlations into Fisher *z* correlations before averaging them.

### Results

In this study, we measured classification images of Mooney faces to investigate whether there are face templates that support holistic face recognition and whether these templates were observer specific. In our experiment, participants classified which stimuli were more face-like.

**Figure 3.**
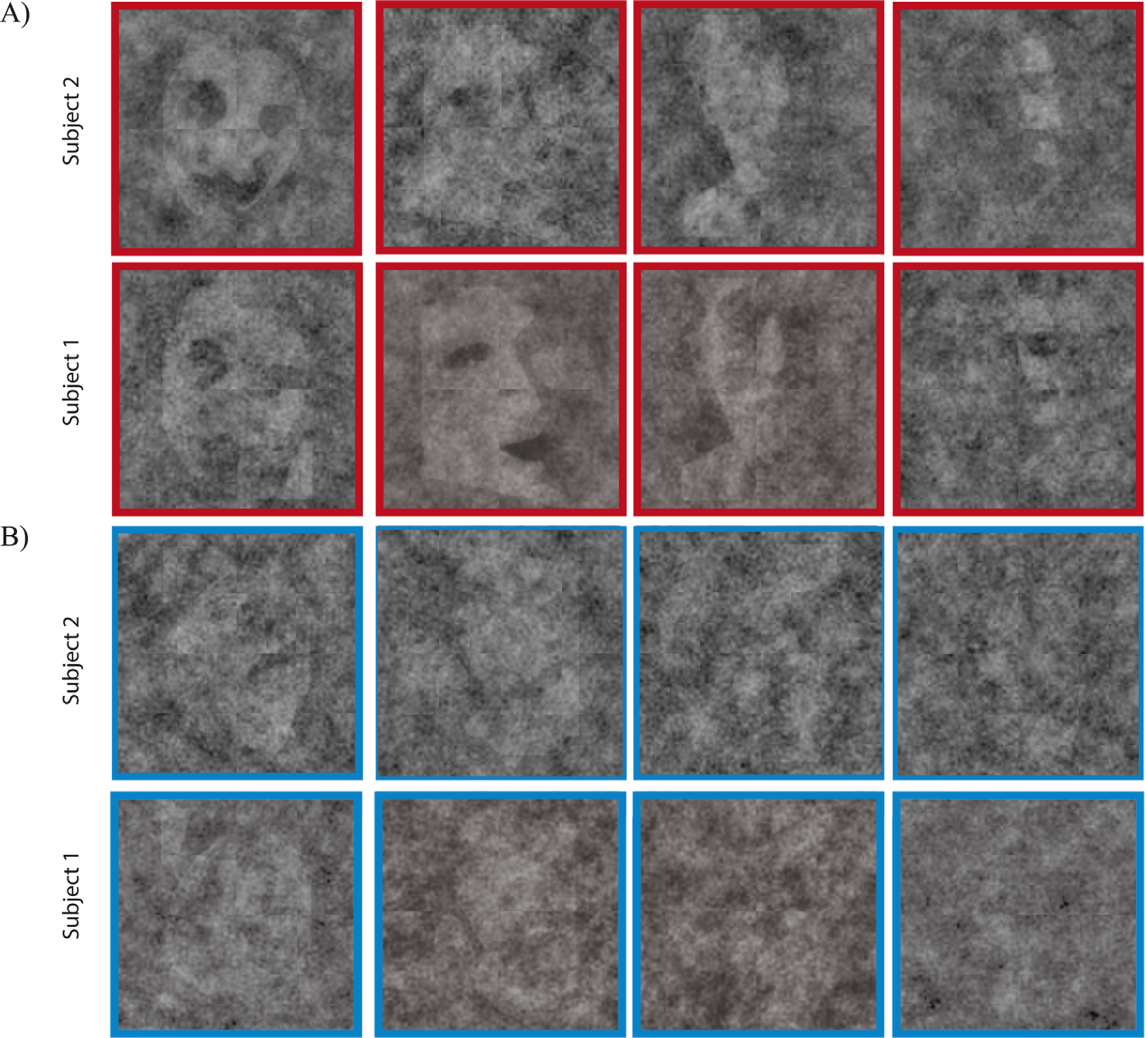
Sample resulting classification images of Mooney faces in Experiment 1 for two subjects. (A) Top row: upright Mooney faces (in red). The Mooney face Classification images are recognizable (albeit noisy and ghostly). (B) Bottom row: inverted Mooney faces (in blue). Inverted Mooney face classification images did not appear to be very obvious. See main text for details.

**Figure 4.**
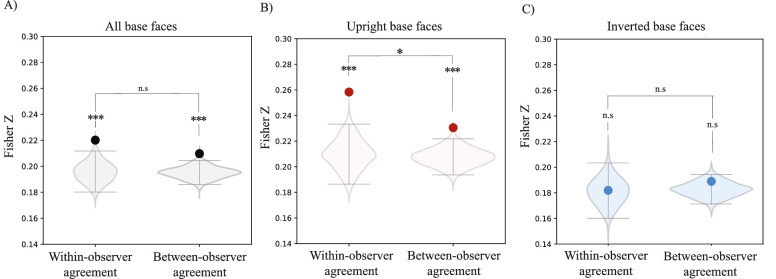
Results of Experiment 1. (A) Within- and between-observer agreement for classification images collapsed across upright and inverted faces. (B) Within- and between-observer agreement for classification images of upright base Mooney faces. (C) Within- and between-observer agreement for classification images of inverted base Mooney faces. In all panels, solid dots represent the empirical correlations, and the violin plots represent the respective permuted null distributions. Error bars represent the upper 97.5% and lower 2.5% boundaries of the permuted null correlation distribution. ***p < 0.001, **p < 0.01, *p < 0.05. Nonsignificant comparisons are indicated by n.s.

The outcome per observer was a classification image for each base face tested (see Figure 3 for some examples). Casual inspection hints that the classification images of upright faces seem to be visible and face-like (Figure 3A). In contrast, classification images of inverted faces appeared to be less recognizable (Figure 3B). Our hypothesis was that an observer's classification images represented the observer's underlying face representations. To test whether classification images were even nonrandom, we collapsed the upright and inverted faces (vertical pairs of faces in Figures 3A, 3B) together. On the one hand, the collapsed within observer correlation was the average of the within observer correlations of upright and inverted classification images. This averaged correlation allowed us to investigate whether, across all base faces, classification images were not random. On the other hand, the collapse between observer correlations was computed as the average of the between-observer correlations of upright and inverted classification images, and it represented the agreement across observers for all faces. Altogether, the goal of this analysis was to check and confirm that we could even measure classification images that were significantly different than random responses. If classification images of face-likeness can be measured, then there should be significant within- and between-subject consistency. We found that there was both a significant within- and between-observer correlation for collapsed classification images of Mooney faces (within: Fisher *Z* = 0.22, *p* < 0.01; between: Fisher *Z* = 0.21, *p* < 0.01; Figure 4A). This result shows that we effectively measured classification images of face-likeness and that they were not due to random responses.

The Mooney faces that we used in this experiment require holistic processing to be recognized (Canas-Bajo & Whitney, 2020; 2022; Farzin et al., 2009; Latinus & Taylor, 2005; McKone, 2004; Moore & Cavanagh, 1998). If recognition of Mooney faces is supported by holistic face templates, then classification images of Mooney faces should reflect an advantage for upright faces. Inverted Mooney faces are hard or even impossible to recognize (Kanwisher, Tong & Nakayama, 1998; McKone, 2004; Latinus and Taylor, 2005), so we expected weaker classification images of inverted Mooney faces. Figure 3 seems to support this division, showing what appear to be weaker classification images for inverted faces. To address this question quantitatively, we computed the within- and between-observer agreement for classification images of upright and inverted faces, separately. We found that there was a significant within-observer agreement in classification images for upright faces (Fisher *Z* = 0.26, *p* < 0.001, per permutation test; Figure 4B) but not inverted faces (Fisher *Z* = 0.18, *p* > 0.05, per permutation test; Figure 4C). A direct comparison of upright and inverted within-observer correlations further confirmed that the day-to-day consistency of the classification images was higher for upright than inverted base faces (*t* = 90.96, *p* < 0.001). That is, only upright Mooney base faces led to non-random, consistent classification images from session to session. Inverted Mooney faces did not. Similarly, we found a significant between-observer agreement only for upright Mooney faces (Fisher *Z* = 0.23, *p* < 0.01; Figure 4B), but not inverted Mooney faces (Fisher *Z* = 0.19, *p* > 0.05; Figure 4C).

One of our primary goals was to investigate whether classification images of Mooney faces were observer specific. To this end, we compared the within- and between- observer agreement in the classification images of upright and inverted faces, separately. We found that within-observer agreement of classification images of upright Mooney faces was significantly higher than the between-observer agreement (*p* < 0.05, per permutation test; Figure 4B). This difference was not significant in classification images of inverted Mooney faces (*p* > 0.05, per permutation test; Figure 4C). This result suggests that there are day-to-day consistent individual differences in the classification images of upright Mooney faces, which require holistic processing.

### Discussion

The results of Experiment 1 indicate that classification images of Mooney faces are consistent from session to session. We also found that there are individual differences in classification images of Mooney faces, specific to upright faces. This result is consistent with the idea that holistic face recognition is supported by matching Mooney faces to face templates (Cavanagh, 1991) and that these templates are idiosyncratic—unique to each individual observer. One concern with Experiment 1 is that data were collected online using the Qualtrics platform, and participants completed the experiment on their own computer monitors. Thus we could not gamma correct or control the linearity of each individual participant's monitor. Consequently, the individual differences found may have been influenced by differences across monitors. It is important to note that our inverted condition served as a partial control for this possibility, and the dissociation in results found for upright and inverted faces suggests that the individual differences found in upright Mooney faces are probably not due to differences in gamma correction across the monitors. Nevertheless, individual differences in monitors are an important concern, as are individual differences in room set up, ambient lighting, monitor distance, and other environmental factors. We therefore conducted a second experiment, in the laboratory, with a single controlled and calibrated monitor. The goal of Experiment 2 was to replicate Experiment 1 results and to further investigate whether the individual differences in classification images reflect an underlying idiosyncrasy in the internal representations of faces.

## Experiment 2

Experiment 2 had two goals. First, to control for a possible contribution of individual differences in monitors, hardware, software settings, environmental factors, or any other differences due to the online nature of the first experiment. We therefore conducted the experiment in-person with a controlled environment, constant hardware, and gamma correction of the monitor used for all subjects. The second, more theoretically important goal of Experiment 2 was to investigate whether there is pattern completion within classification images of Mooney faces. If Mooney faces are recognized through a matching process to stored face templates, then classification images should show that observers fill in missing face feature information within the original Mooney face. Additionally, if holistic face recognition is supported by idiosyncratic templates as suggested by Experiment 1, then we should expect that the way information is filled in is unique to each observer, at least for upright faces.

### Method

#### Participants

Nineteen participants (eight male, eleven female) took part in this experiment. All subjects were undergraduate students at the University of California, Berkeley, and provided written consent forms before participation. All experimental procedures were approved by the UC Berkeley Institutional Review Board.

#### Material

Stimuli were presented on a gamma corrected CRT monitor at 100 Hz refresh rate, with 1024 × 768 pixels resolution and a horizontal screen size of 40.5cm. The monitor was placed 60 cm from a chin rest that stabilized the participant's head. At this distance, all the face stimuli shown during the experiment subtended 6° visual angle. The presentation of the stimuli was controlled using MATLAB R2016b with Psychophysics Toolbox 3 (Brainard and Vision, 1997; Kleiner, Brainard, & Pelli, 2007).

#### Design, procedure and data analysis

Design, procedure, and data analysis was the same as in Experiment 1. Average time between participants’ experimental sessions was 1.7 days (Standard Deviation = 0.8)

### Results

**Figure 5.**
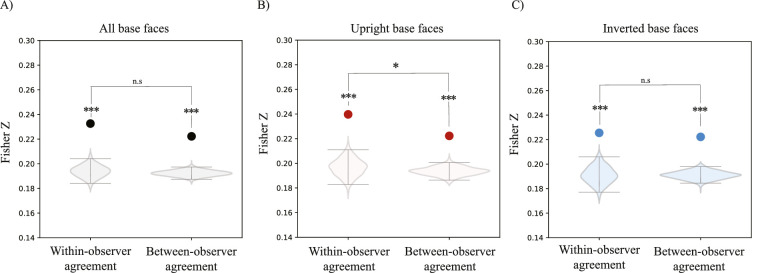
Results of Experiment 2. (A) Within and between-observer agreement for classification images of all upright and inverted base faces collapsed. (B) Within and between-observer agreement for classification images of upright base faces. (C) Within and between-observer agreement for classification images of inverted base faces. In A-B, black dots represent the empirical correlation found and the violin plots represent the respective permuted null correlations. Error bars represent the upper 97.5% and lower 2.5% boundaries of the permuted null correlation distribution. ***p < 0.001, **p < 0.01, *p < 0.05. Nonsignificant comparisons are represented with n.s.

The first goal of Experiment 2 was to replicate Experiment 1’s results with a single gamma-controlled monitor and consistent environment across observers. Consistent with Experiment 1, we found significant within- and between-observer agreement in classification images collapsed across upright and inverted conditions, which further confirms that that classification images of Mooney faces are consistent from session to session and not due to random noise (within correlation: Fisher *Z* = 0.23, *p* < 0.001; between-subject correlation: Fisher *Z* = 0.22, *p* < 0.001; Figure 5A).

**Figure 6.**
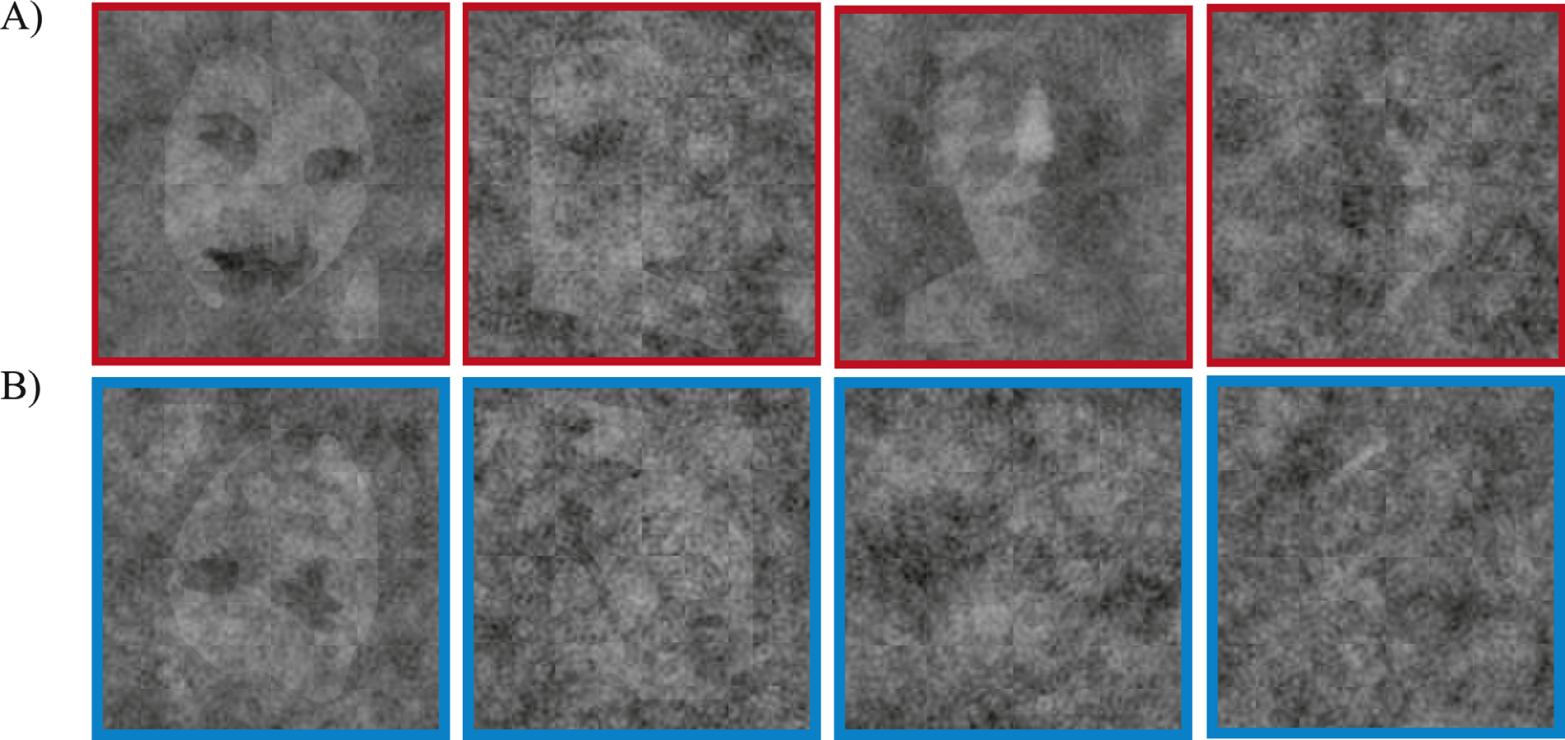
Sample resulting classification images of Mooney faces in Experiment 2. (A) Top row: upright Mooney faces (in red). The Mooney face Classification images are recognizable (albeit noisy and ghostly). (B) Bottom row: inverted Mooney faces (in blue). Inverted Mooney face classification images did not appear to be very obvious. See main text for details.

We next compared the consistency and uniqueness of classification images of upright and inverted faces, separately. As in Experiment 1, classification images of upright faces displayed a significant within- and between-observer agreement (within-observer Fisher *Z* = 0.24, *p* < 0.001; between-observer Fisher *Z* = 0.22, *p* < 0.001; Figure 5B). In this experiment, we found that the within- and between-observer agreement remained significant for inverted faces (within-observer Fisher *Z* = 0.22, *p* < 0.001; between-observer Fisher *Z* = 0.22, *p* < 0.001, per permutation test; Figure 5C), but it was nevertheless weaker than classification images of upright faces (*t* = 26.32, *p* < 0.001). Indeed, as in Experiment 1, casual inspection also suggests that the classification images of upright faces seem to be more visible and face-like (Figure 6A) than classification images of inverted faces (Figure 6B).

Importantly, we found a relatively higher within-observer agreement than between-observer agreement that was specific to upright faces (*p* < 0.05, per permutation test; Figure 5B). Replicating Experiment 1, this finding indicates that there are individual differences in classification images that are specific to upright Mooney faces, which require holistic processing. The individual differences found in Experiment 1 are evidently not due to the lack of a consistent environment across observers. In contrast, classification images of inverted faces were not found to show observer-specific idiosyncrasies (*p* > 0.05, per permutation test; Figure 5C).

**Figure 7.**
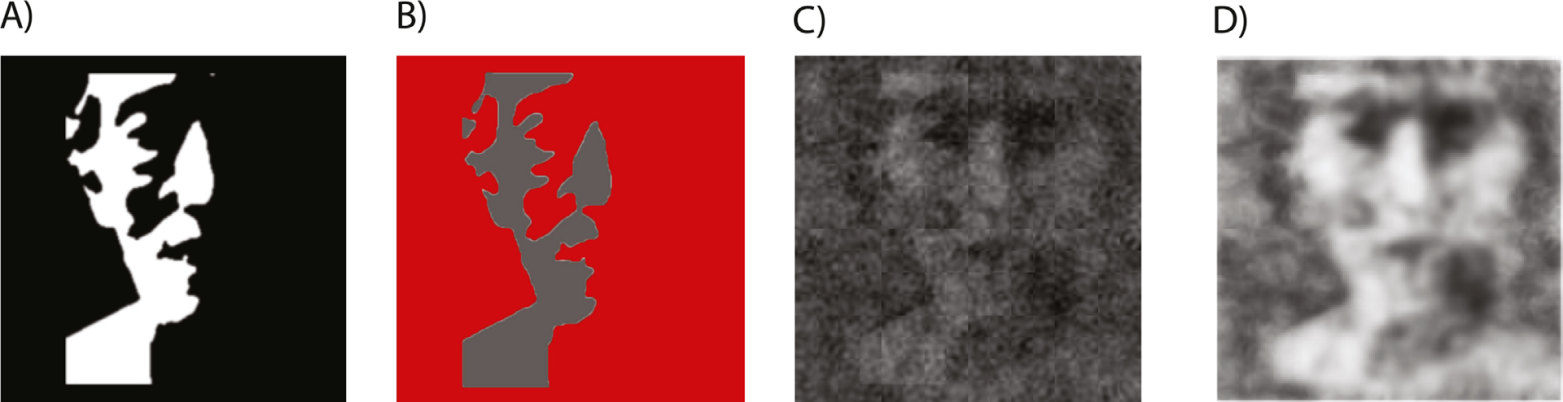
(A) Original Mooney face used as base face. (B) In red, original black areas in classification images. Correlations calculated within these signal-free and otherwise featureless regions to analyze the extent of pattern completion. (C) Classification image reflecting pattern completion. (D) Classification image with enhanced contrast to facilitate visualization of the filled-in areas.

So far, we showed that there are individual differences in classification images of Mooney faces that are consistent session to session. However, the question still remained as to what these day-to-day consistent classification images represent. Our hypothesis was that they reflected participants’ internal representation of faces. Mooney faces do not contain easily parsed or bottom-up segmentable information about the parts of the faces (one has to know it is a face before localizing or identifying a particular eye or nose), and some or all face features can be missing from them (e.g., a half-illuminated faces). If classification images reflect participants’ templates for faces, then they might reveal filled-in information that is missing at the image level. For example, a base Mooney face that is only partially illuminated on the left side, and mostly in complete darkness on the right side (Figure 7A), might nevertheless evoke a classification image that includes details in the invisible portion of the image. To test this hypothesis, we investigated whether there is pattern completion in classification images of Mooney faces. We operationalized pattern completion as the information present in the classification image only within the black regions of the Mooney face (regions highlighted in red in Figure 7B). Any filling-in or pattern completion of information could involve a number of top-down decision processes and may be, essentially, an “expectation” that the observer had about whether the face had two eyes, whether it remained obscured in shadow, whether it had an attached contour corresponding to the cheek, and more. To increase the power of the pattern completion analysis and considering the similarity in results of Experiments 1 and 2, we collapsed participant data from both experiments for the following tests.

**Figure 8.**
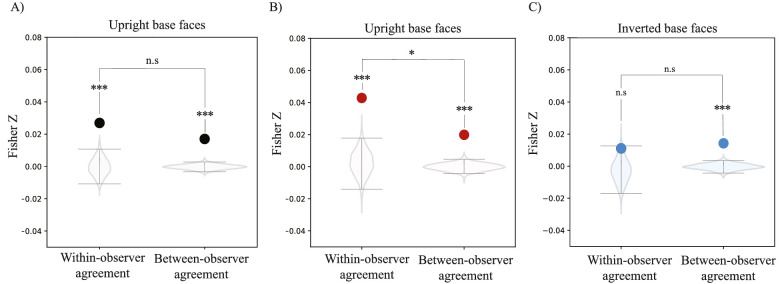
Pattern completion results. (A) Within and between observer agreement in signal-free black areas of all original upright and inverted Mooney faces collapsed. (B) Within and between observer agreement in black areas of the original upright Mooney face. (C) Within and between observer agreement in black areas of the original inverted Mooney face. In all panels, solid dots represent the empirical correlation found and the violin plots represent the respective permuted null correlations. Error bars represent the upper 97.5% and lower 2.5% boundaries of the permuted null correlation distribution. ***p < 0.001, **p < 0.01, *p < 0.05. Nonsignificant comparisons are represented with n.s.

To measure whether classification images showed pattern completion of missing face features within the black regions of the original Mooney face, we calculated the within-observer agreement within these black regions (Figure 8A). These black regions do not contain any signal, so any consistency in the selected noise patterns within these areas would suggest that the observer has perceptually completed parts of the face that were not originally there. We found significant within- and between-observer agreement within the black regions of all collapsed classification images (within-observer correlation: Fisher *Z* = 0.03, *p* < 0.001; between-observer correlation: Fisher *Z* = 0.02, *p* < 0.001; Figure 8A). This result indicates there is pattern completion in classification images of Mooney faces and is consistent with the hypothesis that recognition of Mooney faces is supported by a matching process to stored templates (Cavanagh, 1991).

When we explored pattern completion within classification images of upright and inverted Mooney faces separately, we found a significant within-subject correlation within the black regions of upright Mooney faces (Fisher *Z* = 0.04, *p* < 0.001, per permutation analysis; Figure 8B). This indicates that there was unique filling-in of missing information within single individual observers. Moreover, this pattern completion was consistent from session to session. This confirms that the pattern completion result found in upright Mooney faces was not random; that is, there was meaningful information present in those regions that was not originally there. As a visualization, note a representative observers’ classification image in which pattern completion is evident (Figure 7): even though there is no single face feature information on the right side of the original upright Mooney face (Figure 7A), the classification image of a sample observer showed qualitative completion of the missing features (the right eye, the chin line, the continuation of the mouth, etc.; Figure 7C). Pattern completion becomes more evident visually when we enhance the contrast of the image (Figure 7D). We found no consistent pattern completion for inverted Mooney faces (Fisher *Z* = 0.02, *p* > 0.05; Figure 8C).

Moreover, if observers’ templates that support face recognition are idiosyncratic, then classification images should show unique pattern completion for each participant. We found a significant but relatively low between-observer agreement in the way black regions in upright Mooney faces were filled-in (Fisher *Z* = 0.02, *p* < 0.001; Figure 8B). This indicates some consistency between observers, but the within-observer correlation was significantly higher than the between-observer correlation (*p* < 0.5, permutation analysis; Figure 8B). These results suggest that there is pattern completion in high-level object representations of upright faces, and that the perceptually filled-in information was unique to each observer. Inverted faces were different. Although we found significant between-observer agreement in the pattern completion of inverted Mooney faces (Fisher *Z* = 0.01, *p* < 0.001; Figure 8C), the difference between within- and between-observer agreement was not significant. This result suggests that there is weaker and less idiosyncratic pattern completion for inverted faces.

### Discussion

In Experiment 2, we aimed to replicate the results found in Experiment 1 under a more controlled experiment set up. In sum, we found that classification images of Mooney faces were consistent from session to session, as in Experiment 1. Similarly, we found observer-specific differences in classification images of Mooney faces that were specific to upright faces. This finding confirms that the results of Experiment 1 were not due to a floor effect.

Furthermore, to understand whether Mooney face recognition is supported by matching them to stored face templates, we explored whether there was day-to-day consistent and unique filling-in of missing facial information in classification images. To address this question, we looked at the within- and between-subject agreement only within the black regions of the original Mooney face. We found within-observer agreement within these black regions, indicating that there is pattern completion in high-level object representations. This result is in line with previous research showing that Mooney face perception is supported by matching to stored templates (Cavanagh, 1991; Damasio, Damasio & Van Hoesen, 1982; Sekuler & Abrams, 1968; Sergent, 1984; Simpson & Crandall, 1972; Smith & Nielsen, 1970; Valentine, 1991). Pattern completion was stronger in upright than inverted classification images. Interestingly, the filled-in pattern was unique to each observer only in upright faces, which suggests that the idiosyncrasy in face recognition is specific to holistic processing.

## General discussion

Previous work suggests that face recognition is supported by a matching process between observed faces and internal face templates (Cavanagh, 1991; Damasio, Damasio & Van Hoesen (1982); Sekuler & Abrams, 1968; Sergent, 1984; Simpson & Crandall, 1972; Smith & Nielsen, 1970; Valentine, 1991). Faces that best match those internal face representations are processed more efficiently than those that do not (Rossion, 2008; Rossion & Boremanse, 2008). However, proposed definitions of face templates often describe a representation of low-level features, which do not generally account for holistic processing of faces and the advantage it affords face recognition (Dakin & Watt, 2009; Viola & Jones, 2004). In the present study, we used classification images of Mooney faces to investigate whether there are face templates that are consistent session to session, whether they support holistic processing of Mooney faces, whether they are observer specific, and whether they allow for pattern completion of holistic representations.

Our results show that there are day-to-day consistent classification images of Mooney faces. The reverse-correlation task used here was purely data driven: observers were not biased toward any external definition of face-likeness, so they applied their internal face presentations to classify the stimuli as face-like. Note that unlike face discrimination and identification tasks commonly used in classification image experiments, the face-likeness task used here may also involve detection processes (Tyler, & Chen, 2006). This suggests that observers’ classification images reflected their underlying, implicit face representations. Importantly, we found that classification images of upright Mooney, but not inverted Mooney faces, were observer specific. This is consistent with the idea that there are stored face templates that support holistic processing and that those templates may be idiosyncratic, which could be driving the individual differences in holistic processing found previously in the literature (Canas-Bajo & Whitney, 2020; de Heering & Rossion, 2008; Goodman, Sayfan, Lee, Sandhei, Walle-Olsen, Magnussen, Pezdek, & Arredondo, 2007; Ferguson, Kulkofsky, Cashon & Casasola, 2009; Kuefner, Cassia, Vescovo & Picozzi, 2010; Susilo, Crookes, McKone, & Turner, 2009). These results support the conclusion that holistic and part-based processing are distinct mechanisms of face recognition (McKone, 2004; Moscovitch, Winocur & Behrmann, 1997), and that holistic processing is idiosyncratic, whereas part-based processing is relatively universal (Canas-Bajo & Whitney, 2020).

Furthermore, we found that observers filled in missing information in the original base Mooney face. Humans are highly sensitive to incomplete and partial visual information, resulting in object and scene recognition that proceeds seemingly unimpaired by occlusions and noise. Many examples of this have been reported, including contour and surface-based filling-in and patten completion (Gold, Murray, Bennett, Sekuler, 2000; Sekuler, Gold, Murray & Bennett, 2000). Here, we used Mooney faces as stimuli, which do not contain enough low-level information to be uniquely processed in a part-based manner (Canas-Bajo & Whitney, 2020). The pattern completion results found in the present study cannot be explained by known contour integration, surface filling-in, or feature-based pattern completion processes. Interestingly, we found that the way missing information was filled in was idiosyncratic only in upright Mooney faces. Mooney face recognition may therefore be supported by matching the impoverished images to idiosyncratic stored templates. Future research should investigate whether the filled-in or completed regions correspond to edges of the face (so called “attached-contours,” after discounting shadows) or whether the classification image also reveals cast shadows edges (which are uninformative about the structure of the face; Cavanagh 1991).

Last, we also found weaker but day-to-day consistent classification images of inverted Mooney faces with weaker pattern completion and no idiosyncrasies. The stability of these inverted classification images was less efficient than for upright faces, which confirms that our results were not due to a floor effect in the inverted condition. It also agrees with the idea that the part-based pathway to face recognition may be less efficient (Sekuler, Gaspar, Gold & Bennett, 2004). Mooney faces can occasionally maintain some low-level features and be partially supported by a combination of holistic and part-based processing (Canas-Bajo & Whitney, 2020). This suggests that face templates may not only support holistic processing of upright faces but also part-based processing of inverted faces and perhaps object recognition more generally. This is in line with previous work that reported templates for objects (Biederman & Bar, 1999; Rock & DiVita, 1987; Poggio & Edelman, 1990; Logothetis, Pauls, Bülthoff, & Poggio, 1994; Tarr, 1995; Tarr & Bülthoff, 1995).

Our results show that classification images may reveal information about internal stored templates that are unique to each observer. However, it is worth noting that the classification images we measured may have been influenced by nonlinear and decision-related processes that cannot be discerned from our results (Murray, 2011; Neri, 2018). Future research should quantify to what extent an observer’s behavior can be predicted from their unique classification image and to what extent a classification image captures aspects of visual processing that significantly influence an observer’s trial-by-trial task behavior. Nevertheless, our results showed that there are significant individual differences in observers’ classification images, which suggest that they may reveal unique information about the templates that observers putatively use to recognize Mooney faces.

A long history of computer vision research has aimed to reproduce the template-matching theory of face recognition. Turk and Pentland (1991) defined a computational face recognition system in which faces are encoded as vectors of weights, called eigenfaces, and stored in memory. Eigenfaces are a low-cost representation of faces. When the system encounters a novel face, recognition occurs through a matching procedure to the stored eigenfaces—a type of template. Others have developed Deep Convoluted Neural Networks models of face recognition that highlight the need for internal representations to be general and flexible, so that they can achieve the level of expertise with familiar and unfamiliar faces that we see in humans (Blauch, Behrmann & Plaut, 2021; Orru, Marcialis & Roli, 2020), parallel to the well-known advantage for familiar faces. This and other computer-vision–based face recognition research suggests that models that use template matching can, at least in principle, reproduce the patterns of human face recognition.

Future research can use our approach to investigate the origin and development of face templates at the observer level. The proposed approach of studying holistic perception using reverse correlation classification image method is powerful and could be especially useful in narrowing and identifying the neural mechanisms of holistic face perception. Unfortunately, it is a very time-consuming method, but it can be done within individual observers, as our data suggests. Future work might therefore be able to use the technique in combination with electroencephalography, functional magnetic resonance imaging, and magnetoencephalography. The technique might also be useful for evaluating the biological plausibility and correspondence between face identification algorithms and holistic face perception by assessing classification images in both domains.

Altogether, our results provide evidence that classification images can be used as an unbiased method to tap into observer-specific internal representations of faces. Furthermore, the present study provides a new method for future research to investigate the nature of face templates, such as understanding how many templates are required to support our expert face recognition system (Laurence, Baker, Proietti & Mondloch, 2021); investigating the plasticity of face templates over development (de Heering & Rossion, 2008; Laurence, Baker, Proietti & Mondloch, 2021); or making predictions from an observer's classification image of which faces will be more efficiently processed.
